# Variation of Chemical Composition and Antioxidant Properties of Kiwiberry (*Actinidia arguta*) in a Three-Year Study

**DOI:** 10.3390/molecules28010455

**Published:** 2023-01-03

**Authors:** Piotr Latocha, Barbara Łata, Paweł Jankowski

**Affiliations:** 1 Department of Environmental Protection and Dendrology, Institute of Horticultural Sciences, Warsaw University of Life Sciences—SGGW, Nowoursynowska 166, 02-787 Warsaw, Poland; 2 Section of Basic Research in Horticulture, Institute of Horticultural Sciences, Warsaw University of Life Sciences—SGGW, Nowoursynowska 166, 02-787 Warsaw, Poland; 3 Department of Computer Information Systems, Institute of Information Technology, Warsaw University of Life Sciences—SGGW, Nowoursynowska 166, 02-787 Warsaw, Poland

**Keywords:** seasonal changes, cultivar, phenolics, ascorbate, carotenoids, total antioxidant capacity, weather conditions, minerals, computer analysis

## Abstract

The quality of fruit as a source of bioactive ingredients is related to the genetic characteristics of plants, but it can also be modified by growing conditions. Therefore, long-term research can be extremely valuable in evaluating various crop plants, especially novel ones. The aim of the research was to test four popular European kiwiberry (*Actinidia arguta*) cultivars (‘Geneva’, ‘Bingo’, ‘Weiki’, ‘Anna’) in terms of selected morphological features, yield, and chemical composition as well as their variability over 3 years. It can be concluded that the studied genotypes were very diverse in terms of the biochemical compounds’ concentration in individual seasons. The cultivars ‘Anna’ and ‘Weiki’ were the most similar ones with respect to each other in terms of morphology and chemical composition. The cultivars ‘Bingo’ and ‘Geneva’ were definitely different. ‘Bingo’ was characterized by the largest and most uniform fruits in each season and had the highest concentration of vitamin C but the lowest carotenoid concentration. In turn, ‘Geneva’ produced the smallest fruit in each season with the highest concentration of polyphenols and a high concentration of carotenoids and displayed the highest antioxidant capacity regardless of the determination method. The research was performed with the application of computer-supported statistical analysis.

## 1. Introduction

The fruit’s internal quality is one of the most important criteria determining interest in the species/cultivar of future producers and consumers alike. Internal quality traits are determined by a group of phytochemicals with different functions and activities [1]. The high dynamics of the bioactive compound metabolism is related to their function for the plants themselves as signaling and protective molecules in response to biotic and abiotic stress [2]. To a large extent, the concentration of bioactive compounds is determined by the genetic factor, but external factors, such as soil physicochemical properties, weather conditions during fruit development and ripening, and even the way of plantation running (plant management and pruning method), may also have a strong influence. The fruit’s chemical composition between growing seasons may vary within wide limits [3]. The analysis of the impact of soil environment characteristics and climatic conditions is particularly difficult due to their complexity.

Kiwiberry is still a new, promising species with a growing cultivation area worldwide [4,5]. The resistance of kiwiberry plants to low temperatures in winter, due to which they can be grown in countries with cooler climates where conditions do not allow the cultivation of “fuzzy” kiwifruit, is one of their main advantages [6]. Compared with the commonly known kiwifruit, kiwiberry fruits are about 10 times smaller; have delicate, edible skin; and can be eaten whole, and hence serve as a “convenient snack”. Containing over 20 different bioactive compounds in chemical structure and capacity, kiwiberry is commonly considered the most nutrition-dense fruit and presented as a healthy fruit or functional food [7,8]. Kiwiberry’s prohealth values have been the subject of many studies. Kiwiberries have a protective effect on the cardiovascular [9] and digestive systems and exhibit antiglycemic, antihypertensive, and anticholinergic activities, and also after processing [10]. Kiwiberries, unlike kiwifruit, have a relatively short storage potential and limited transportability and so are usually eaten locally in the area where they are produced, which in effect reduces carbon footprint [11]. In recent years, interest has also increased in by-products from the production of kiwiberry as it was found to be a rich source of bioactive compounds used in the manufacture of food additives [12] or cosmetics [13].

As previously mentioned, the chemical composition of the fruit can be strongly modified by environmental factors [14]. In the case of kiwiberry, there is still a limited amount of data on the range of variability in the fruit chemical composition and yield in the context of climate change, notably the instability of weather conditions in consecutive years and extreme weather events. Most of the available reports on this species and various factors affecting fruit quality are based on the analysis of one growing season [15,16]. Hence, the fruit stability in terms of the bioactive compound concentration, including the cultivar context recommended for cultivation, is relatively inadequately recognized. Knowledge of these is no doubt important in recommending cultivars for cultivation or for breeding purposes.

Therefore, the aim of this research carried out over 3 years was to assess the stability of the chemical composition and antioxidant capacity of the four most popular *Actinidia arguta* cultivars in Europe: ‘Geneva’, ‘Bingo’, ‘Weiki’, and ‘Ananasnaya’ (abbreviation: ‘Anna’). It was also an attempt to link them with selected morphological and physiological parameters.

## 2. Results and Discussion

### 2.1. The Weather Conditions during the Experimental Seasons

**Figure 1:**
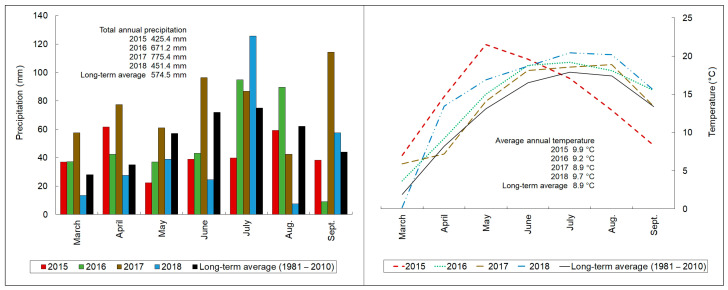
Data on the sum of monthly rainfall (**left graph**) and average temperature (**right graph**) in examined growing seasons at the experimental site in relation to the long-term average.

The weather conditions in 2015, 2016, 2017, and 2018 differed compared with long-term averages (Figure 1). All in all, the driest March–September season was recorded in 2015, and subsequently in 2018. In both years, the total precipitation was below 80% of the mean one observed between 1981 and 2010. In the 2018 season, the cumulative rainfall was over 115% of the long-term mean. In the 2017 season, the highest precipitation was recorded. The distribution of precipitation in each year was not even. The 2015 season could be divided into three periods: wet March–April, dry May–August, and average August–September. Similarly, the four periods in 2016 were: wet March–April, dry May–June, wet July–August, and very dry September. In 2017, wet March–June and dry August–September were observed. The strongest rainfall variability in 2018 could be summarized in four phases: dry March–June, very wet July, very dry August, and wet September. In all the experimental seasons, the average temperatures were higher than the long-term means. In the years 2016, 2017, and 2018, they were constantly above the long-term averages (apart from April 2017 and March 2018). In comparison with the 2016 and 2018 seasons, two periods could be distinguished in the 2015 season: hot March–June and cold July–September. Strong spring frost on 17 April and 10 May destroyed young shoots, resulting in no yield this season.

### 2.2. The Fruit and Plant Morphology and Its Year-to-Year Variability

The examined cultivars differed in the average berry weight, which ranged from 6.8 g (‘Geneva’) to 9.1 g (‘Bingo’) (Table 1). The two other cultivars—‘Weiki’ and ‘Anna’—produced medium-sized fruits of similar average weight. The differences in the yield results were much less distinct than in the case of the average fruit weight, partly due to much higher variances of the mean results for both years and cultivars (Table 1). The highest average fruit yield was obtained from vines of the ‘Anna’, ‘Weiki’, and ‘Geneva’ cultivars, 41.0, 42.0, and 42.1 kg/plant, respectively. The ‘Bingo’ cultivar, characterized by the biggest fruits, had the lowest average yield, 28.7 kg/plant, on average. Still, because of the high variances, the only significant difference was noticed between ‘Bingo’ and ‘Weiki’.

Apart from the influence of the genetic differences between the cultivars, the size of the fruit and the final yield per plant are determined by the number of flower buds formed in spring and the pollination effectiveness. As the vines in each experimental block and pollinators did not vary, the variation of the flower bud formation and the pollination effectiveness may be related to changing weather conditions during and before each season. That, however, was beyond the scope of this study.

Significant differences in fruit weight between seasons were observed. On average, the largest fruit was harvested in 2016, and the smallest in 2015. The mean fruit weight in 2018 was slightly higher than in 2015 but with the largest variance between samples. Apart from differences in seasonal flower bud formation and pollination effectiveness, such ordering was probably due to the differences in weather conditions observed in the summer months of July and August, the period of intensive fruit growth. In the 2016 season, July and August were rainy and warm, providing very good conditions for fruit growth. In contrast, July in 2015 was dry, and both July and August in 2015 were relatively cold. Finally, the year 2018 was characterized by warm summer but a very wet July and an extremely dry August.

In the case of 2015 and 2016, the years with ‘stable’ weather in the summer months, the difference between the mean weights of the fruit of individual cultivars was the same as the discussed average differences. For each cultivar, the fruit weights were higher in favorable 2016 than in adverse 2015 (in the case of ‘Bingo’, the difference was not statistically significant due to the high variability of fruit size in 2016). The highest between-cultivar variability was recorded in 2018. The average weight of ‘Geneva’ fruit was the smallest, that of ‘Weiki’ and ‘Anna’ medium, and that of ‘Bingo’ the biggest (same as in 2016) in that year, in comparison with the fruit of the same cultivars collected in other years.

During three seasons of research, ‘Bingo’ (two homogeneous groups) was the most stable one in terms of fruit size, while each of the other cultivars formed three homogenous groups. Moreover, the lowest seasonal average ‘Bingo’ fruit weight equaled 91% of the highest weight, while the lowest kiwifruit weights amounted to only 71%, 73%, and 76% for ‘Geneva’, ‘Weiki’, and ‘Anna’, respectively.

The largest average yield was obtained in 2018. The harvests in 2015 and 2016 did not differ significantly, though in 2016 when the biggest fruit was collected, the total yield was numerically lowest during all the experimental years. The highest average yield per plant for each cultivar was recorded in 2018. Apart from ‘Bingo’, the next to the biggest yield was collected in 2015. It was statistically indistinguishable from the year 2016 for ‘Anna’ and ‘Geneva’, and in the case of ‘Weiki’, that yield was not significantly different from 2018. Again, ‘Bingo’ was the most stable cultivar, giving the most similar yield in all three seasons (no statistical differences between years were observed), and the lowest yield accounted for 61% of the largest. In comparison, the lowest harvests amounted to only 35%, 43%, and 49% of the highest ones recorded for ‘Geneva’, ‘Weiki’, and ‘Anna’, respectively.

The average area of the leaf blades of the cultivars studied varied between 4573 mm^2^ (‘Bingo’) and 6973 mm^2^ (‘Geneva’) (Table 1). The ‘Geneva’ cultivar had the significantly largest average leaf blade area compared with other cultivars, the leaves of which were of similar statistically undistinguishable size. In light of the obtained results, the size of the leaves of individual cultivars of *Actinidia* appears to have a genetic basis and was not modified significantly by weather conditions. No statistical differences in leaf blade area for each cultivar in subsequent years of this study were observed (Table 1).

The lowest average concentration of chlorophyll was found in leaves of the ‘Bingo’ cultivar (17.1 a. u.). It was at a higher and even on the same level (22.3–24.3 a. u.) in the other cultivars. All growing seasons differed significantly as regards the mean leaf chlorophyll concentration. Irrespective of cultivars, the highest chlorophyll concentration in leaves was measured in 2018 and 2016, and the lowest one in 2015. Except for ‘Geneva’, other cultivars achieved similar chlorophyll concentrations in leaves in 2015 and 2016.

### 2.3. The Fruit Chemical Composition, Antioxidant Capacity, and Its Year-to-Year Variability

Kiwiberry fruit is considered to be a rich source of bioactive compounds with significant prohealth effects, as was confirmed by many earlier studies [8,15,17]. Similar to *A. chinensis* var. *deliciosa* and *A. eriantha*, the most important bioactive compounds in *A. arguta* fruit consist of phenolic compounds, ascorbate, thiol compounds, pigments, and minerals [18,19,20].

Global phenolic concentrations calculated as gallic acid equivalents (GAE) ranged from 685 (‘Weiki’) to 1222 (‘Geneva’) mg GAE/kg FW and differed in individual seasons, which is clearly visible in high SD values for global cultivar effect (Table 1). Fruit sampled in the 2015 and 2016 growing seasons exhibited significantly more phenolic compounds than those harvested in 2018. That was probably due to the more favorable weather conditions in that season. ‘Geneva’ was characterized by the highest concentration of polyphenols each year. Our findings confirm previous results showing that ‘Geneva’ is richer in polyphenols than other popular cultivars grown in Europe [17]. Among the cultivars tested, ‘Bingo’ expressed the greatest variation and lower concentration of phenolics depending on the growing seasons. There may be several reasons for this. The first is the greater sensitivity of this cultivar genetically. Second, the concentration of polyphenols in the fruit may be related to their size (skin-to-flesh ratio) because, according to previous studies, the concentration of polyphenols in the kiwiberry peel is much higher than in the pulp [21,22]. Finally, a higher concentration of phenolic compounds in fruits, regardless of the genetic factor, is usually associated with stress factor occurrence, of biotic and/or abiotic nature, in plant surroundings [2].

The average ascorbate concentration ranged from 552 mg/kg FW (‘Weiki’) to 796 mg/kg FW (‘Bingo’) (Table 1). Three cultivars (‘Anna’, ‘Geneva’, ‘Weiki’) accumulated a similar amount of ascorbate, and in contrast to them, ‘Bingo’ fruit exhibited significantly higher ascorbate concentration. As in the case of phenolic compounds, the fruits of most cultivars accumulated the least ascorbate in the 2018 season. The average concentration of ascorbate in fruit in the 2015 and 2016 seasons did not differ significantly. ‘Anna’ and ‘Weiki’ were the most stable ones in seasonal ascorbate concentration, while ‘Bingo’ the most variable (Table 1). Ascorbate concentration, similar to phenolics, has a stress background and can be explained similarly as in the case of polyphenol concentration. The negative impact of some environmental conditions, mainly high temperatures and the period of its occurrence, on the concentration of vitamin C and carbohydrates was observed in previous studies on kiwifruit [23]. Our results, independent of seasonal fluctuations, confirm the previous opinion that *A. arguta* is a valuable source of vitamin C [15,16].

In kiwiberry fruit, 1.69–3.26 mg/kg FW L-cysteine and 145–208 mg/kg FW total glutathione (tGSH) concentrations were recorded depending on the cultivar (Table 1). The ‘Geneva’ and ‘Anna’ cultivars were the richest sources of glutathione, while ‘Bingo’ and ‘Weiki’ had a significantly lower concentration of this tripeptide. Smaller differences were observed in the case of L-cysteine, where its concentration was significantly higher in ‘Anna’, while the other cultivars constituted one homogeneous group in terms of this compound concentration. With the exception of ‘Geneva’, the differences in glutathione concentration in individual years for the tested cultivars reflect the average concentrations recorded in the years studied.

Significant amounts of carotenoids and chlorophylls were also determined in kiwiberry fruit. On average, for three seasons of research in fruits, 1.25 mg (‘Bingo’)—2.69 mg (‘Geneva’)/kg FW lutein and 1.67 mg (‘Bingo’)—2.86 mg (‘Weiki’)/kg FW beta-carotene were determined (Table 2). In addition, 9.12 mg (‘Bingo’)—20.0 mg (‘Geneva’)/kg FW of chlorophyll a and 3.10 mg (‘Bingo’)—7.32 mg (‘Weiki’)/kg FW chlorophyll b were also found (Table 2). The highest average concentration of both carotenoids and chlorophylls was found in the fruit of the ‘Weiki’ and ‘Geneva’ cultivars, followed by ‘Anna’. An almost two times lower concentration of carotenoids and chlorophylls was recorded in the ‘Bingo’ fruit. Similar cultivar differences in carotenoids and chlorophyll concentrations in *A. arguta* were found by Hishiyama et al. [24]. According to these authors, kiwiberry fruit is one of the richest sources of lutein among commonly consumed fruits. Overall, the growth conditions in 2016 favored the accumulation of carotenoids and chlorophylls. Similar (LUT, Chl_b) or much lower (B-car, chl_a), compared to 2016, concentrations were obtained in 2015. The cultivars tested, especially the ‘Anna’ and ‘Weiki’ fruits, were characterized by having a relatively stable level of lutein in the years examined. In the case of other pigments, their concentration varied strongly depending on the cultivar and the year of the study. Similarly to TPC, the lowest concentration of pigments was found in all cultivars in the 2018 season. This was probably due not only to milder weather conditions in 2018, but also to the lack of yield in 2017, and thus to lower plant loads and their overall better condition.

One of the most frequently used measures for assessing the fruit’s health-promoting value is the ability of its extracts to scavenge free radicals. In order to elucidate the potential differences in fruit antioxidant capacity as fully as possible in this study, the determinations were carried out using three tests (DPPH, ABTS, FRAP). The antioxidant capacity of kiwiberry extracts on average, depending on the cultivar, stood at 18.7–28.9 mmol TE/kg (ABTS), 19.3–24.4 mmol TE/kg (DPPH), and 18.0–28.9 mmol TE/kg (FRAP). The fruit of the ‘Geneva’ cultivar was characterized by the highest antioxidant potential, although the results did not differ significantly from those for the ‘Bingo’ (Table 2). Irrespective of the test used, the ‘Weiki’ cultivar was characterized by the lowest antioxidant capacity, although against ABTS and DPPH radicals, similar results were obtained for ‘Anna’. The mean results of antioxidant capacity determinations in individual research seasons were distributed in a diversified and not very logical manner (Table 2). When analyzing individual seasons and cultivars, the highest capacity measured by the ABTS method was shown by all cultivars in the 2016 season, and by the FRAP method in the 2015 season. In turn, the DPPH analysis divided the cultivars tested in this respect as ‘Anna’ and ‘Bingo’ had the highest results in 2018, and ‘Geneva’ and ‘Weiki’ in 2015. The significant differentiation obtained was probably related, among others, to the different structures of phenolic compounds in kiwiberry and the diversity of the remaining antioxidant compounds, directly related to their different antioxidant capacity. A large variation of antioxidant capacity between seasons is visible in high SD values for the global cultivar effect, similar to the concentration of phenolics (Table 1 and Table 2). These results may suggest a more complicated influence of individual groups of bioactive compounds, and even individual compounds, the concentration of which changed in each season, on individual methods of antioxidant capacity determination. It is well known that various substances occurring in plant organisms show a different ability to scavenge free radicals; therefore, their changing concentration in fruits strongly modifies the results of determinations by individual methods. The weather conditions could influence not only quantitative but also qualitative changes in characteristics of the internal quality of kiwiberry fruit as confirmed in our previous multiseason studies [25] and proven in the case of apples [26].

The variation of the macro- and microelement concentrations in the cultivars under study was smaller compared with bioactive compound concentrations, although in some cases, significant differences between cultivars and research seasons were also found (Table 3 and Table 4). The dominant macronutrients consisted of potassium (0.95–1.27% DW), nitrogen (0.75–0.93% DW), and calcium (0.30–0.37% DW) as well as micronutrients—iron (21.6–35.2 mg/kg DW), boron (17.3–17.6 mg/kg DW), and manganese (8.8–15.4 mg/kg DW).

These findings coincide with the results of other studies [18,27]. The fruits of the ‘Bingo’ cultivar contained significantly less P, K, Cu, and Mn, while ‘Geneva’ was distinguished by the highest concentration of calcium, and ‘Weiki’ of iron. Such differences in mineral composition between kiwiberry cultivars were also described by Gralak et al. [28]. The authors indicate ‘Weiki’ as being the most abundant one in the analyzed microelements. Additionally, the Bieniek and Dragańska [29] research demonstrated that the macroelement concentration in some Ukrainian cultivars depends on weather conditions during specific phenophases. In the 2015 season, all cultivars showed the highest accumulation of calcium and magnesium, but the lowest of potassium. On the other hand, the 2018 season was characterized by the highest concentration of nitrogen (except for ‘Bingo’) and phosphorus in fruits of all cultivars.

The concentration of micronutrients in fruit was less regularly distributed. The highest concentration of iron, zinc, and manganese (except for ‘Geneva’) was found in the 2015 season, and that of copper and boron (except for ‘Bingo’) in the 2018 season. Overall, the 2016 season was characterized by a lower concentration of minerals in the fruit than the other growing seasons. In the case of the concentration of Fe, Ca, and Zn, there were no interactions between the cultivar and the harvest year. The variability of the concentration of these minerals and Mn was weak compared with that of other characteristics. On average, the concentration of macronutrients in the fruits of the cultivars studied in three research seasons was found in the following order: K > N > Ca > P > Mg and microelements Fe > B > Mn > Zn > Cu.

It can be concluded that the studied genotypes were most diverse in terms of the biochemical compounds’ concentration in individual seasons, which is clearly visible in the high SD values for the global cultivar effect, especially for phenolics (Table 1). The cultivars ‘Anna’ and ‘Weiki’ were the most similar to each other in terms of morphology and chemical composition—somewhere between the other two cultivars and exhibiting the lowest antioxidant capacity. The characteristics of the ‘Anna’ and ‘Weiki’ cultivars in the 2018 season were clearly different from that in the other two seasons. The cultivars ‘Bingo’ and ‘Geneva’ were definitely different. The first was characterized by the largest and most uniform fruits in each season with the highest concentration of vitamin C but the lowest carotenoid concentration. In turn, ‘Geneva’ produced the smallest fruit in each season with the highest concentration of polyphenols and a high concentration of carotenoids as well as the highest antioxidant capacity, regardless of the method of determination. These features of ‘Geneva’ confirm the previously obtained results of research on this cultivar [9,17,25]. Despite many biochemical differences, it can be seen that smaller fruits were characterized by the highest antioxidant capacity. Regardless of the genetically determined concentration of antioxidants in a given cultivar, that could be related to the higher peel-to-pulp ratio. Similar conclusions were reached by Gündüz et al. [30] in their research on blueberry.

Plants are complex organisms in which all the body elements influence each other. That may apply to the biochemical components as well as morphological features. The plant feature that has a significant impact on the vigor and rate of physiological processes and thus on the concentration of bioactive compounds is the size of the leaf blades and their chlorophyll concentration [31]. The concentration of chlorophyll in leaves and the accumulation of macro- and micronutrients in fruits, apart from the genetic factor, may also be influenced by agrotechnical factors, such as mineral fertilization, mainly nitrogen nutrition [32,33]. The obtained results show that genetic factors and conditions of growing seasons play a key role in the accumulation of bioactive compounds in the kiwiberry fruit. Moreover, a significant interaction also takes place between the cultivar and the conditions during the growing season, and relationship differs from compound to compound. In practice, this means greater or lesser variability in the health properties of a given cultivar in subsequent years. Therefore, multiseason research in order to assess the stability of cultivars in terms of the concentration of bioactive compounds in fruits of various plant genotypes is essential for the selection of the most valuable cultivars. One-year experiments can often lead to wrong conclusions.

### 2.4. Interrelationships between Chemical Components and Physical and Physiological Properties

**Figure 2:**
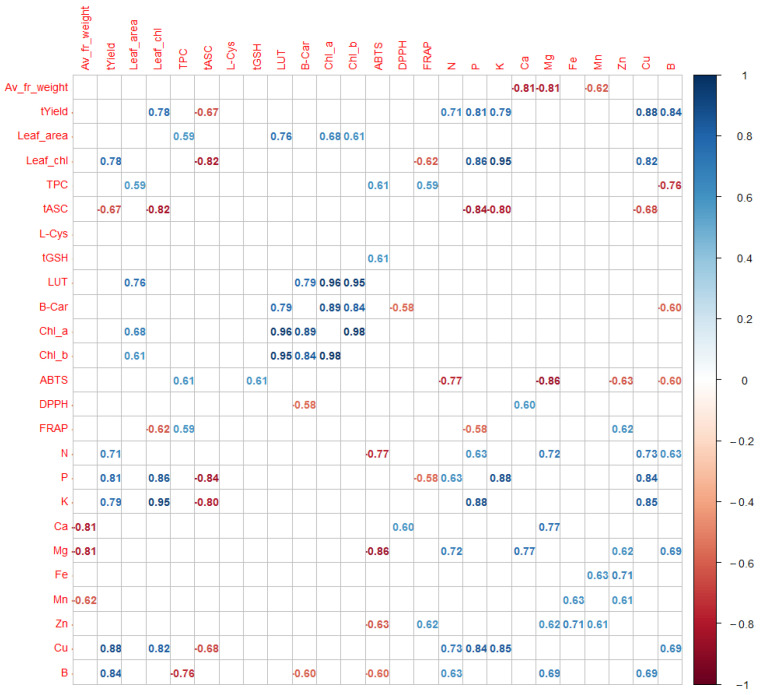
A graph of the Pearson correlation matrix (correlogram) of the morphological, chemical, and antioxidant properties of the fruits. The significance level is equal to 0.05. The blue color represents the positive correlations, and the red color the negative ones. Each number shows a value of significant correlation. The color intensity represents the correlation strength, according to the legend.

**Figure 3:**
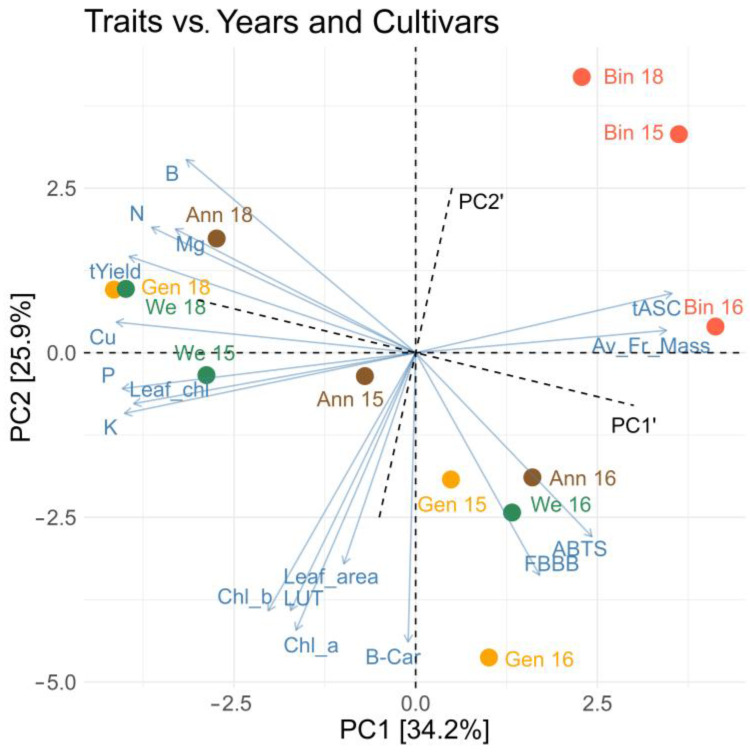
PCA biplot (PC1 vs. PC2) for four cultivars examined in 3 years of the experiment and their morphological, chemical, and antioxidant properties. Only the traits with high correlation to PC1 and PC2 (at squared cosine greater than 0.6) are presented.

**Figure 4:**
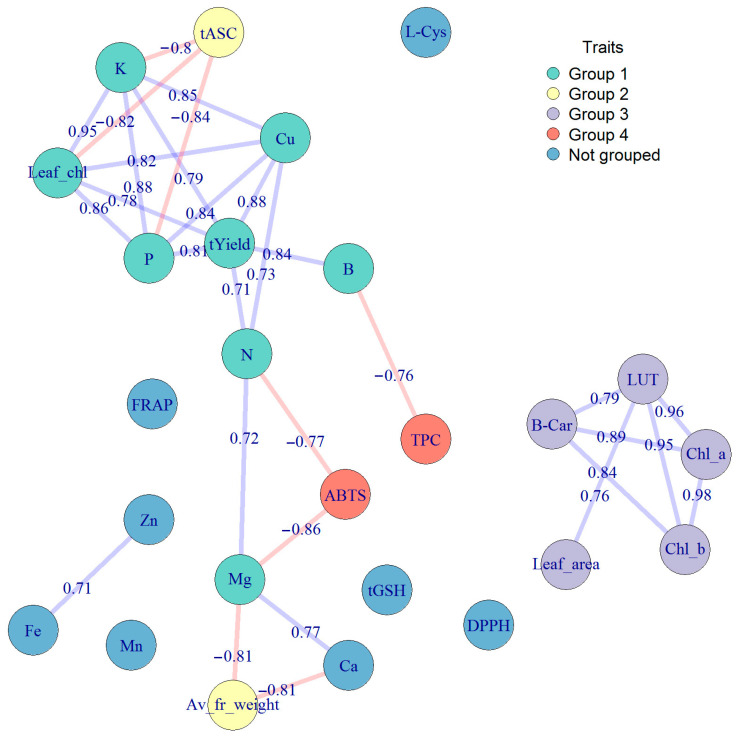
The strongest (with values above 0.7 or below −0.7, significance level of 0.05) correlations between members of the described groups in the plot were obtained using correlation-based network analyses. Colors define groups of the morphological, chemical, and antioxidant properties of the fruits related to each other, as described in the manuscript.

The analyses of relations between chemical components, morphological features, and antioxidant capacity of kiwiberry were performed using Pearson correlation and PCA. Figure 2 shows a correlogram of all significant relations between the examined attributes at the level of significance of 0.05 (the correlations at the level of significance 0.1 are presented in Supplementary Figure S1). The results proved a very complex and multilateral influence of various plant and fruit attributes on each other. As can be seen in Figure 2, the content of ascorbate was negatively correlated with the height of yield and the content of chlorophyll in leaves, and the content of polyphenols, lutein, and chlorophyll in fruits was positively correlated with the leaf blade area. Moreover, it is interesting that some minerals were strongly positively correlated with each other (e.g., Zn with Mg, Fe, Mn, or Cu with N, P, K). The PCA plot (see Figure 3) explains about 60% of sample variability and allowed us to distinguish four groups of the examined attributes. Finally, Figure 4 presents the strongest (with values above 0.7 or below −0.7, significance level of 0.05) correlations between members of the described groups in the plot obtained using correlation-based network analyses.

**Figure 5:**
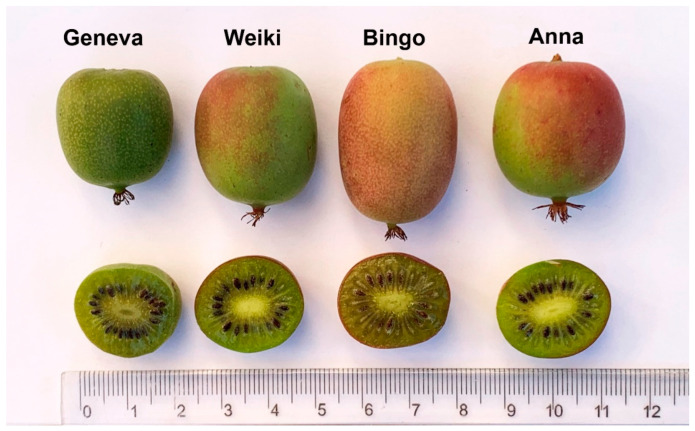
The fruit appearance of the *A. arguta* cultivars used in the study.

According to the analysis, the first group of traits (Group 1) consisted of the macronutrients N, P, and K and Mg, Cu, and B micronutrients, Leaf_chl, and yield. Group 2 was formed by the ascorbate concentration (tASC) and the average fruit weight, which, according to network plots (Figure 3 and Figure 5), were not significantly correlated. Groups 1 and 2 were most strongly associated with the PC1 component, which accounted for approximately 34% of the variability between the tested samples of four kiwiberry cultivars over 3 years. These are two opposite groups: the increase in the concentration of ascorbate and the average fruit weight was accompanied by a decrease in the concentration of the mentioned micro- and macroelements and a decrease in yield. Let us note that some of these relations are significant at a level below 0.1—see Supplementary Figure S1.

Chlorophyll is the primary pigment used in photosynthesis [34]. It is, therefore, natural that its concentration within the kiwiberry plant leaves is positively correlated with the total fruit yield. A similar correlation between chlorophyll concentration and grain yield has been observed before in different cultivars of wheat, barley, and oat [35] and maize [36] and aromatic rice [37].

A study by Smith et al. [38] showed that the annual uptake of nutrients by mature kiwi vines is greatest for nitrogen (N), potassium (K), and calcium (Ca), followed by chlorine (Cl), phosphorus (P), magnesium (Mg), sulfur (S), and micronutrients. This set contains the N, P, and K macronutrients and Mg from the first group of the kiwiberry traits. Nitrogen, phosphorus, and potassium fertilizers are the three main plant nutrients used worldwide [39]. It has been shown that they are also important components of kiwifruit vines needed for increasing yields [40]. It is commonly known that nitrogen and phosphorus are essential macronutrients for plant growth and development. Potassium, together with magnesium, is required to synthesize chlorophyll and critically contribute to the process of photosynthesis and the subsequent long-distance transport of photoassimilates [41]. Copper is required in the process of photosynthetic electron transport [42]. Finally, the foliar application of boron has been observed to increase crops of such fruits as sour cherry, hazelnut, and almonds [43].

Hence, the increase in the concentration of the N, P, K, Mg, and Cu elements in the kiwiberry plants should lead to an increase in the chlorophyll concentration of the leaves and the total fruit yield. Such a relation may further explain a proportional increase in the concentration of the nutrients in the kiwiberry fruit, though that should be examined in further studies.

The number of fruits is usually negatively correlated with the average fruit size and weight [44]. Crop load is therefore usually reduced via pruning and thinning. Though a higher crop load leads to a decline in average fruit weight, it may still bring a greater total yield due to an increased number of fruits [45]. In such a case, the correlation between the average fruit weight and yield is negative, as observed for kiwiberry in the presented study (at a significance level of 0.1). A similar relation has been noticed for kiwi fruit, for instance, by Testolin [46]. The decrease in fruit weight should additionally lead to an increased concentration of micro- and macronutrients. That can be another reason for the positive relation between nutrients contained within Group 1 and the total yield of fruit.

As can be seen in the correlation diagram (Figure 4), the ‘center’ of Group 1 consisted of the P, K, and Cu concentrations and the yield. The average values of these features can be arranged according to the ‘Weiki’, ‘Anna’, ‘Geneva’, and ‘Bingo’ cultivars, with differences between the successive cultivars being statistically significant or insignificant, depending on the feature, with the distinction of the ‘Bingo’ cultivar, which represented the lowest values of each of these features. In the case of features included in Group 1, the average values in the years can be arranged as follows: 2018, 2015, 2016 (and 2018, 2016, 2015 only in the case of Leaf_chl.). As can be seen in the graph in Figure 2, this general order of years was repeated in each of the tested cultivars. The ascorbate concentration was opposite to that in Group 1, while the average fruit weight was significantly the highest in 2016 and the lowest in 2015. The smallest between summer variability in terms of the features from Groups 1 and 2 is shown by ‘Bingo’.

According to the PCA and correlation-based network analyses, Group 3 of the examined traits concentrated on the concentration of carotenoids (LUT and beta-carotene), chlorophylls (Chl_a and Chl_b), and the size of the leaves. Group 4 consisted of AA (ABTS radicals) and phenolics (TPC). These groups were most strongly associated with the PC2 component, which accounted for approximately 26% of the variability between the samples tested. However, they are also related to the PC1 component, negatively Group 3 and positively Group 4, which explains the lack of strong correlations between these groups of features. Looking at the additional rotated axes PC1 and PC2, it can be seen that features from Group 3 are arranged orthogonally to features from Groups 1 and 2; that is, they are not related to them. Similarly, Group 4 features lie along the PC1 axis more opposite the plot center than Group 1 features. This illustrates their significant negative correlations with the B, N, and Mg concentrations. The positive correlation between the concentrations of chlorophyll and carotenoids has been also reported for other crop species, such as kale [47], Swiss chard [48], lettuce [49], and peas [50]. In the case of apples, it has been shown that the green cultivars had the highest pigment concentration in both the peel and the flesh, which were followed in decreasing order by the peel of some red-skinned cultivars and the flesh of the yellow ones [51]. The general ranking of the attributes included in Group 3 by kiwiberry cultivar was as follows: ‘Geneva’ (with pure green skin), ‘Weiki’ and ‘Anna’ (with slightly reddish skin), ‘Bingo’ (with the most reddish skin and slightly yellowish flesh), and by the years 2016, 2015, and 2018.

The AA (ABTS) and polyphenol concentration (Group 4) were similar due to the cultivars ‘Geneva’, ‘Bingo’, ‘Anna’, and ‘Weiki’ but different due to the years. This explains the weaker correlation between these features. The rest of the examined attributes showed weaker links with other features.

## 3. Materials and Methods

### 3.1. Chemicals

2,2-Azino-bis (3-ethylbenzthiazoline-6-sulfonic acid) (ABTS), 1,1-diphenyl-2-picrylhydrazyl (DPPH), 6-hydroxy-2,5,7,8-tetramethylchroman-2-carboxylic acid (Trolox), FBBB, lanthanum (III) chloride heptahydrate, FeCl_3_·6H_2_O, CuCl_2_·2H_2_O, and 2,9-dimethyl-1 and 10-phenanthroline (neocuproine) were purchased from Sigma Chemical Co., St. Louis, MO, USA. Chlorophyll a and b, lutein and β-carotene standards, and 2,4,6-tripyridyl-s-triazine (TPTZ) were purchased from Fluka Chemie, Buchs, Switzerland. All reagents were of analytical grade. Deionized and distilled water was used throughout.

### 3.2. Experimental Design, Plant Material and Weather Conditions

The research was carried out in the 2015, 2016, and 2018 growing seasons (severe spring frosts in 2017 severely damaged the developing shrubs, and no yield was achieved). The research was carried out on fully fruiting kiwiberry plants (4-, 5-, and 7-year-old vines) in a commercial plantation in Bodzew, Mazovian Voivodeship, in central Poland (6B USDA Plant Hardiness Zone). The habitat conditions and the plantation methods were described in detail in our earlier publication [20]. Therefore, briefly, the vines on the plantation grew 4 × 4 m apart, were guided by T-bar supports, and were pruned in the standard way for this form of guidance [52]. The research covered four cultivars of kiwiberry, important in commercial cultivation in Europe and morphologically different, namely, ‘Geneva’ (an early cultivar with green fruit), ‘Bingo’ (a cultivar with medium ripening time and elongated fruit with a pink blush), ‘Weiki’, and ‘Ananasnaya’ (‘Anna’) (late ripening time, oval fruit with a red blush) (Figure 5). The experiment was set up in a random block design. Each of the cultivars was represented by 3 blocks of 4 vines each. At the beginning and the end of each block, buffer vines were applied, followed by male plants (pollinators). The vines in each block were of similar size and vigor for each cultivar. The vines grew on soil characterized by approximately 1.8% of organic matter concentration, and pH in the soil suspension in distilled water was in the range of 6–6.5. Fertilization was performed on the basis of a chemical analysis performed in the spring of each growing season in accordance with recommendations applicable to berry plants [53]. The rows were kept free of weeds in herbicide fallow.

The Davis Vantage Pro2 Plus meteorological station (Davis Instruments, Hayward, CA, USA) set up on the plantation was used to record the temperature and rainfall. Comparative long-term averages for this region come from the years 1981–2010.

### 3.3. Plant Morphological and Chemical Measurements

The leaf area was measured with the Digishape ver. 1.9 computer program (Cortex Nova 2005, Poland). Ten fully developed leaves from terminating shoots (July) were collected from each block. The leaves were scanned immediately after harvesting, and the scan was measured (only leaf blades). The leaf area was averaged over a block and reported in mm^2^. In order to establish the yield of each cultivar, all fruits from each block were harvested separately and then weighed (total yield). After harvest, the fruit of each cultivar was weighed to record the total yield per replication (block). The yield was expressed in kg per vine (plant). The chlorophyll concentration in the leaves was measured five times during the growing season at intervals of about 3 weeks, starting as of the end of May, using the CL-1 apparatus from Hansatech Instruments Ltd., Norfolk, UK, and expressed in agreed units (a. u.). At each time of sampling, 20 leaves from each treatment’s replication were measured, and the result was averaged. For each cultivar, 60 measurements were made on each date (delivered from 3 blocks of 20 measurements each). The result for each cultivar in consecutive growing seasons was expressed as an average of all measurements in that season.

### 3.4. Fruit Sample Preparation and Measurements

The fruits of each cultivar were harvested at the harvest maturity stage (firm fruit with black seeds, 6.5°–7° Brix). The ‘Geneva’ fruit was collected first (at the beginning of September), followed by ‘Bingo’, and finally by the ‘Weiki’ and ‘Anna’ cultivars (in mid-September). Fruit weight determination was performed on 30 fruits for each cultivar in the season (3 repetitions of 10 fruits for each cultivar). Every repetition covered similar-sized fruits free of any damage or deformations. The result is presented as the average weight of individual fruit +/− standard deviation (SD). For further analysis, harvested and sorted fruit was stored in a cold chamber (temperature of 1 °C and 90% RH) for 3 weeks, and then for a few days at 20 °C until eating maturity (soft fruit, ~18° Brix) was reached. Afterward, 60 similar-sized fruits with no damage (3 repetitions of 20 fruits for each cultivar) were selected for chemical analysis. Next, the fruit was frozen in liquid nitrogen and transferred to a deep freeze at −80 °C until analysis.

### 3.5. Determination of Bioactive Compounds

The concentrations of ascorbate (L-AA + DHAA, reduced and oxidized forms, respectively), thiol compounds (total glutathione, e.g., GSH and GSSG, reduced and oxidized forms, respectively, and L-cysteine), and carotenoids (lutein and β-carotene) were determined by using high-performance liquid chromatography (HPLC), as described in detail in our previous study [20]. The results were expressed in mg per kilogram on a fresh weight (FW) basis.

The total phenolic concentration (TPC) was measured after two-step extraction with methanol in an ultrasonic bath [54] using Fast Blue BB (4-benzoylamino-2,5-dimethoxybenzenediazonium chloride hemi[zinc chloride] salt, FBBB) reagent, a new method recently developed by Medina [55] and described there in detail. The results were expressed as gallic acid equivalents (GAE) in mg/kg FW.

### 3.6. Antioxidant Capacity Measurements

Antioxidant capacity was determined with three different tests: FRAP (ferric reducing antioxidant power), ABTS (2.2′-azino-bis-3-ethylbenzothiazoline-6-sulfonic acid), and DPPH (1,1-diphenyl-2-picrylhydrazyl) assay, as described in detail by Łata [56], Re et al. [57], and Brand-Wiliams et al. [58], respectively. The results were calculated using a calibration curve and expressed as millimole Trolox equivalents (TE) per kilogram of fresh weight (mmol TE/kg FW).

### 3.7. Determination of Fruit Macro- and Micronutrients

Fruit samples (10 fruits per cultivar and repetition) were washed, oven-dried at 70 °C, and ground to a fine powder. Dry weight was recorded after drying at 105 °C. The concentrations of macronutrients (N, P, K, Ca, and Mg) and micronutrients (Cu, Fe, Mn, Zn, and B) were conducted in an accredited laboratory, the Chemical and Agricultural Station in Warsaw (Poland, Mazowieckie Voivodeship, accreditation number AB 312, http://www.oschr-warszawa.pl, accessed on 5 July 2021), which meets the requirements of the PN-EN ISO/lEC 17.025:2005 standard. Standard procedures were used to analyze the total nutrient concentrations in fruit tissues. The plant material was mineralized using H_2_SO_4_ and H_2_O_2_. N concentration was determined by the Kjeldahl method. Phosphorus concentration was determined at a 470 nm wavelength with a spectrophotometric method using ammonium molybdate, HNO_3_, and ammonium metavanadate. Flame atomic absorption spectroscopy (FAAS) at 766.5, 285.2, and 422.7 nm was used to determine the K, Mg, and Ca concentrations, respectively. The results were expressed as a percentage of dry weight (% DW). Flame atomic absorption spectroscopy (FAAS) at 324.8, 248.3, 279.5, and 213.9 nm was used to determine the Cu, Fe, Mn, and Zn concentrations, respectively. The boron concentration was evaluated by inductively coupled plasma optical emission spectrometry (ICP-OES) at 249.678 nm. The results were expressed as mg/kg of dry weight (DW).

### 3.8. Statistical Analysis

The histograms and plots presenting the monthly precipitation and temperature at the experimental site during the years under study, in relation to the long-term average, were prepared in Microsoft Excel 2019.

The repeated measure ANOVA was applied to assess the effect of the cultivar, the year, and their interactions on tested fruit components. The cultivar was considered a between-subjects factor and the year as a within-subjects factor. The analysis was performed separately for each of the examined fruit parameters. Assumptions of sphericity, homogeneity of covariances, and equality of variances were assessed using Box’s M, Mauchly’s, and Levene’s tests, respectively. The data for each combination of cultivar and the year were visually examined for symmetry and lack of outliers. When significant interactions between the factors were observed, the effect of year was analyzed separately for each cultivar. The homogenous groups of years and cultivars were established via pairwise comparisons with Tukey adjustment for the familywise error rate. The Pearson correlations between the examined fruit parameters were presented in a graph of correlation matrix (correlogram) and a network plot. The correlations were computed for the data averaged for each combination of the year and cultivar. Principal component analysis was carried out to graphically summarize the dispersion of morphological, chemical, and antioxidant properties of the examined kiwiberry cultivars in 3 years of the experiment and the interaction between them. The above analyses were performed in the R program version 4.2.1 [59].

## 4. Conclusions

The results of the research and analyses carried out lead to the following conclusions: Kiwiberry (*A. arguta*) is a valuable source of different bioactive ingredients and can be considered a healthy fruit. ‘Bingo’ has the biggest fruit and the lowest crop among the examined cultivars. Importantly, both the average fruit weight and the yield per plant are most stable in the case of ‘Bingo’. The fruit weight varies by only 10% in comparison with about 30% for other cultivars, and the lowest yield constitutes about 60% of the largest in comparison with 35–50% for other cultivars. The overall biocapacity of the kiwiberry cultivars is ‘Geneva’ > ‘Bingo’ > ‘Anna’ > ‘Weiki’. Both the genetic characteristics of the cultivars and weather conditions strongly modify the chemical composition and antioxidant capacity of kiwiberry, which resulted in its high year-to-year differences. The morphological features of plants influence the chemical composition and antioxidant properties of the fruit to a lesser extent than weather conditions. Multiseason studies show to what extent fruit quality can change from one growing season to the next, as well as how much these changes can depend on the cultivar. This information is valuable both for the cultivar selection for production purposes and for breeding.

## Figures and Tables

**Table 1:** Variation in selected morphological and chemical *A. arguta* plant and fruit traits. Data are means (±standard deviation—SD, n = 3) in the subsequent years depending on the cultivar.

Group	Average Fruit Weight (g)	Total Yield (kg/plant)	Leaf Blade Area (mm^2^)	Leaf Chlorophyll Concentration (a. u.)	Fruit Chemical Composition			
					Phenolic Compounds(TPC)	Ascorbate (tASC)	Thiol Compounds	
							L-Cys	tGSH
					mg/kg FW			
Geneva								
2015	6.5 ± 0.1 ^b^	27.8 ± 7.6 ^b^	7196 ± 83	18.7 ± 1.9 ^c^	1462 ± 21 ^a^	750 ± 134 ^a^	2.04 ± 0.03 ^a^	197 ± 2 ^a^
2016	8.1 ± 0.4 ^a^	25.4 ± 3.7 ^b^	7333 ± 687	23.8 ± 3.3 ^b^	1353 ± 190 ^a^	650 ± 68 ^a^	1.34 ± 0.19 ^c^	219 ± 9 ^a^
2018	5.8 ± 0.3 ^c^	72.8 ± 7.0 ^a^	6389 ± 984	28.0 ± 3.6 ^a^	851 ± 96 ^b^	484 ± 25 ^b^	1.69 ± 0.10 ^b^	208 ± 1 ^a^
Weiki								
2015	6.9 ± 0.2 ^c^	40.7 ± 6.8 ^a^	5102 ± 282	21.6 ± 0.9 ^b^	815 ± 174 ^a^	610 ± 63 ^a^	2.00 ± 0.71 ^a^	113 ± 26 ^c^
2016	9.5 ± 0.7 ^a^	25.6 ± 3.8 ^b^	5259 ± 263	22.6 ± 1.6 ^b^	873 ± 74 ^a^	561 ± 81 ^a^	2.31 ± 0.01 ^a^	189 ± 14 ^a^
2018	7.9 ± 0.2 ^b^	60.1 ± 19.2 ^a^	5281 ± 432	28.8 ± 2.5 ^a^	367 ± 76 ^b^	484 ± 18 ^a^	2.15 ± 0.36 ^a^	151 ± 6 ^b^
Bingo								
2015	8.6 ± 0.8 ^b^	21.0 ± 4.7	4371 ± 217	15.8 ± 2.5 ^b^	681 ± 133 ^b^	986 ± 56 ^a^	1.48 ± 0.33 ^c^	117 ± 14 ^c^
2016	9.4 ± 1.4 ^ab^	30.6 ± 7.5	5020 ± 429	16.1 ± 1.4 ^ab^	1305 ± 137 ^a^	758 ± 69 ^b^	2.56 ± 0.15 ^a^	174 ± 25 ^a^
2018	9.4 ± 0.5 ^a^	34.4 ± 8.7	4329 ± 182	19.5 ± 0.8 ^a^	374 ± 95 ^c^	643 ± 16 ^c^	2.01 ± 0.24 ^b^	145 ± 19 ^b^
Anna								
2015	6.7 ± 0.1 ^c^	33.1 ± 3.5 ^b^	4722 ± 31	20.0 ± 1.4 ^b^	1157 ± 54 ^a^	552 ± 84	5.11 ± 0.34 ^a^	178 ± 5 ^c^
2016	8.9 ± 0.3 ^a^	29.6 ± 2.0 ^b^	5050 ± 434	22.0 ± 1.0 ^ab^	1093 ± 132 ^a^	656 ± 30	1.41 ± 0.19 ^c^	227 ± 7 ^a^
2018	7.2 ± 0.3 ^b^	60.4 ± 9.1 ^a^	4950 ± 823	24.8 ± 2.3 ^a^	372 ± 82 ^b^	575 ± 21	3.26 ± 0.23 ^b^	202 ± 6 ^b^
Global cultivar effect (2015, 2016, and 2018)								
Geneva	6.8 ± 1.1 ^C^	42.0 ± 23.8 ^AB^	6973 ± 746 ^A^	23.5 ± 4.8 ^A^	1222± 302 ^A^	628 ± 139 ^B^	1.69 ± 0.32 ^B^	208 ± 14 ^A^
Weiki	8.1 ± 1.2 ^B^	42.1 ± 18.2 ^A^	5214 ± 302 ^B^	24.3 ± 3.7 ^A^	685 ± 261 ^C^	552 ± 76 ^B^	2.15 ± 0.42 ^B^	151 ± 36 ^B^
Bingo	9.1 ± 0.9 ^A^	28.7 ± 8.6 ^B^	4573 ± 423 ^B^	17.1 ± 2.3 ^B^	787 ± 424 ^BC^	796 ± 158 ^A^	2.02 ± 0.52 ^B^	145 ± 30 ^B^
Anna	7.6 ± 1.0 ^BC^	41.0 ± 15.4 ^AB^	4907 ± 488 ^B^	22.3 ± 2.6 ^A^	874 ± 386 ^B^	594 ± 66 ^B^	3.26 ± 1.52 ^A^	202 ± 22 ^A^
Year effect								
2015	7.2 ± 0.9 ^C^	30.6 ± 9.0 ^B^	5347 ± 1158	19.0 ± 2.7 ^C^	1029 ± 332 ^A^	724 ± 191 ^A^	2.66 ± 1.54 ^A^	151 ± 41 ^C^
2016	9.0 ± 0.9 ^A^	27.8 ± 4.7 ^B^	5665 ± 1089	21.1 ± 3.6 ^B^	1156 ± 232 ^A^	656 ± 91 ^A^	1.91 ± 0.58 ^C^	202 ± 26 ^A^
2018	7.6 ± 1.4 ^B^	56.9 ± 17.8 ^A^	5237 ± 974	25.3 ± 4.4 ^A^	491 ± 230 ^B^	547 ± 72 ^B^	2.28 ± 0.65 ^B^	177 ± 32 ^B^
Interaction	<0.05	<0.01	Ns	<0.05	<0.01	<0.001	<0.001	<0.05

TPC: total phenolic concentration expressed in gallic acid (GAE) equivalents; tASC (total ascorbate): L-AA + DHAA; tGSH (total glutathione): GSH + GSSG; L-Cys: L-cysteine; a. u.: agreed units. In each subcolumn, data marked with different lowercase or uppercase superscript letters differ significantly at *p* ≤ 0.05 according to the post hoc pairwise comparisons with Tukey adjustment for the familywise error.

**Table 2:** Variation in *A. arguta* antioxidant concentration and capacity depending on cultivar and growing seasons. Data are means (±SD, n = 3) obtained in the subsequent years depending on the cultivar.

Component	Fruit Pigment Composition				Antioxidant Capacity		
	Lutein (LUT)	βeta-Carotene (B-Car)	Chlorophyll_*a* (Chl_a)	Chlorophyll_*b* (Chl_b)	ABTS	DPPH	FRAP
mg/kg FW					mmol TE/kg FW		
Geneva							
2015	2.99 ± 0.37 ^a^	2.62 ± 0.38 ^b^	20.34 ± 2.46 ^b^	7.58 ± 0.78 ^a^	21.7 ± 1.7 ^b^	35.5 ± 2.8 ^a^	47.3 ± 1.4 ^a^
2016	3.12 ± 0.18 ^a^	3.69 ± 0.05 ^a^	24.67 ± 1.32 ^a^	8.01 ± 0.79 ^a^	43.0 ± 5.5 ^a^	19.2 ± 4.0 ^b^	21.4 ± 3.6 ^b^
2018	1.97 ± 0.02 ^b^	1.70 ± 0.07 ^c^	14.95 ± 0.15 ^c^	5.47 ± 0.04 ^b^	21.5 ± 2.3 ^b^	31.9 ± 3.1 ^a^	17.9 ± 1.8 ^c^
Weiki							
2015	2.72 ± 0.20	2.75 ± 0.09 ^b^	20.16 ± 0.99 ^a^	7.86 ± 0.16 ^a^	12.2 ± 2.4 ^b^	24.4 ± 1.4 ^a^	27.5 ± 4.6 ^a^
2016	2.51 ± 0.09	3.44 ± 0.04 ^a^	20.09 ± 0.85 ^a^	7.17 ± 0.10 ^ab^	28.6 ± 1.8 ^a^	11.0 ± 2.3 ^b^	14.5 ± 1.6 ^b^
2018	2.45 ± 0.09	2.40 ± 0.06 ^b^	18.57 ± 0.20 ^b^	6.93 ± 0.13 ^b^	15.4 ± 1.2 ^b^	22.4 ± 1.2 ^a^	12.0 ± 0.3 ^b^
Bingo							
2015	1.31 ± 0.06 ^a^	1.44 ± 0.10 ^b^	9.12 ± 0.52 ^ab^	3.31 ± 0.15 ^a^	15.1 ± 0.9 ^c^	21.9 ± 0.5 ^b^	35.6 ± 2.6 ^a^
2016	1.52 ± 0.02 ^a^	2.26 ± 0.05 ^a^	11.10 ± 0.26 ^a^	3.70 ± 0.13 ^a^	39.8 ± 3.3 ^a^	21.8 ± 2.9 ^b^	24.0 ± 0.8 ^b^
2018	0.92 ± 0.03 ^b^	1.30 ± 0.02 ^b^	7.13 ± 0.27 ^b^	2.27 ± 0.07 ^b^	20.3 ± 2.5 ^b^	29.4 ± 3.2 ^a^	15.9 ± 2.5 ^c^
Anna							
2015	1.96 ± 0.03	2.65 ± 0.27 ^b^	16.12 ± 0.48 ^b^	6.07 ± 0.01 ^a^	16.1 ± 0.4 ^b^	17.3 ± 0.8 ^b^	39.6 ± 0.7 ^a^
2016	2.03 ± 0.25	3.10 ± 0.05 ^a^	19.09 ± 0.86 ^a^	6.95 ± 0.34 ^a^	33.7 ± 5.4 ^a^	17.1 ± 3.2 ^b^	17.7 ± 2.6 ^b^
2018	1.82 ± 0.05	2.25 ± 0.03 ^c^	14.45 ± 0.53 ^b^	5.12 ± 0.22 ^b^	16.5 ± 2.4 ^b^	24.8 ± 3.1 ^a^	11.6 ± 0.9 ^c^
Global cultivar effect (2015, 2016, and 2018)							
Geneva	2.69 ± 0.59 ^A^	2.67 ± 0.88 ^A^	20.0 ± 4.4 ^A^	7.02 ± 1.30 ^A^	28.8 ± 11.1 ^A^	28.9 ± 7.9 ^A^	28.9 ± 14.1 ^A^
Weiki	2.56 ± 0.17 ^A^	2.86 ± 0.46 ^A^	19.6 ± 1.0 ^A^	7.32 ± 0.44 ^A^	18.7 ± 7.7 ^C^	19.3 ± 6.4 ^C^	18.0 ± 7.6 ^C^
Bingo	1.25 ± 0.27 ^C^	1.67 ± 0.45 ^B^	9.1 ± 1.8 ^C^	3.10 ± 0.65 ^C^	25.1 ± 11.5 ^AB^	24.4 ± 4.4 ^AB^	25.1 ± 8.8 ^AB^
Anna	1.94 ± 0.15 ^B^	2.67 ± 0.39 ^A^	16.6 ± 2.1 ^B^	6.05 ± 0.82 ^B^	22.1 ± 9.2 ^BC^	19.7 ± 4.4 ^BC^	22.9 ± 12.8 ^B^
Year effect							
2015	2.25 ± 0.71 ^A^	2.37 ± 0.60 ^B^	16.4 ±4.9 ^B^	6.21 ± 1.91 ^A^	16.3 ± 3.8 ^C^	24.8 ± 7.1 ^A^	37.5 ± 7.8 ^A^
2016	2.29 ± 0.63 ^A^	3.12 ± 0.56 ^A^	18.7 ± 5.2 ^A^	6.46 ± 1.75 ^A^	36.3 ± 6.9 ^A^	17.3 ± 4.9 ^B^	19.4 ± 4.3 ^B^
2018	1.79 ± 0.58 ^B^	1.92 ± 0.46 ^C^	13.8 ± 4.3 ^C^	4.94 ± 1.77 ^B^	18.4 ± 3.2 ^B^	27.1 ± 4.6 ^A^	14.3 ± 3.1 ^C^
Interaction	<0.001	<0.001	<0.001	<0.001	Ns	<0.001	<0.001

In each subcolumn, data marked with different lowercase or uppercase superscript letters differ significantly at *p* ≤ 0.05 according to the post hoc pairwise comparisons with Tukey adjustment for the familywise error.

**Table 3:** Macronutrients in *A. arguta* fruit. Main effects of two-way ANOVA. Data present means (±SD, n = 3) obtained in the subsequent years depending on the cultivar.

Group	Macronutrients (% DW)				
	N	P	K	Ca	Mg
Geneva					
2015	0.71 ± 0.06	0.16 ± 0.01 ^b^	1.04 ± 0.04 ^b^	0.38 ± 0.04	0.10 ± 0.01
2016	0.34 ± 0.11	0.18 ± 0.00 ^b^	1.17 ± 0.05 ^b^	0.35 ± 0.03	0.07 ± 0.01
2018	1.19 ± 0.17	0.22 ± 0.01 ^a^	1.47 ± 0.04 ^a^	0.39 ± 0.04	0.10 ± 0.01
Weiki					
2015	1.45 ± 0.20	0.21 ± 0.03 ^a^	1.20 ± 0.14 ^ab^	0.38 ± 0.02	0.10 ± 0.01
2016	0.67 ± 0.11	0.17 ± 0.01 ^b^	1.19 ± 0.05 ^b^	0.24 ± 0.03	0.06 ± 0.00
2018	1.52 ± 0.25	0.22 ± 0.03 ^a^	1.41 ± 0.12 ^a^	0.36 ± 0.03	0.10 ± 0.01
Bingo					
2015	0.90 ± 0.68	0.12 ± 0.02 ^b^	0.93 ± 0.06	0.33 ± 0.01	0.09 ± 0.01
2016	0.69 ± 0.11	0.16 ± 0.01 ^a^	0.95 ± 0.02	0.26 ± 0.04	0.06 ± 0.01
2018	0.67 ± 0.11	0.17 ± 0.01 ^a^	0.97 ± 0.12	0.30 ± 0.07	0.09 ± 0.01
Anna					
2015	0.97 ± 0.06	0.17 ± 0.01	1.16 ± 0.04	0.35 ± 0.01	0.10 ± 0.01
2016	0.67 ± 0.19	0.19 ± 0.02	1.27 ± 0.13	0.27 ± 0.05	0.06 ± 0.01
2018	1.15 ± 0.23	0.21 ± 0.01	1.29 ± 0.17	0.29 ± 0.04	0.10 ± 0.01
Global cultivar effect (2015, 2016, and 2018)					
Geneva	0.75 ± 0.38 ^B^	0.19 ± 0.3 ^B^	1.23 ± 0.20 ^A^	0.37 ± 0.04 ^A^	0.09 ± 0.02 ^A^
Weiki	1.22 ± 0.44 ^A^	0.20 ± 0.03 ^A^	1.27 ± 0.15 ^A^	0.32 ± 0.07 ^AB^	0.09 ± 0.02 ^AB^
Bingo	0.75 ± 0.37 ^B^	0.15 ± 0.02 ^C^	0.95 ± 0.07 ^B^	0.30 ± 0.05 ^B^	0.08 ± 0.02 ^B^
Anna	0.93 ± 0.26 ^AB^	0.19 ± 0.02 ^AB^	1.24 ± 0.12 ^A^	0.30 ± 0.05 ^B^	0.09 ± 0.02 ^AB^
Year effect					
2015	1.01 ± 0.42 ^A^	0.17 ± 0.01 ^B^	1.08 ± 0.13 ^B^	0.36 ± 0.03 ^A^	0.10 ± 0.01 ^A^
2016	0.59 ± 0.19 ^B^	0.18 ± 0.01 ^B^	1.14 ± 0.14 ^B^	0.28 ± 0.05 ^B^	0.06 ± 0.01 ^B^
2018	1.13 ± 0.36 ^A^	0.21 ± 0.01 ^A^	1.29 ± 0.23 ^A^	0.33 ± 0.06 ^A^	0.10 ± 0.01 ^A^
Interaction	Ns	<0.05	<0.05	Ns	Ns

In each subcolumn, data marked with different lowercase or uppercase superscript letters differ significantly at *p* ≤ 0.05 according to the post hoc pairwise comparisons with Tukey adjustment for the familywise error.

**Table 4:** Micronutrients in *A. arguta* fruit. Main effects of two-way ANOVA. Data present means (±SD, n = 3) obtained in the subsequent years depending on the cultivar.

Group	Micronutrients (mg/kg DW)				
	Fe	Mn	Zn	Cu	B
Geneva					
2015	24.8 ± 0.9	13.3 ± 1.7	9.97 ± 1.68	5.42 ± 0.51 ^b^	15.5 ± 0.6
2016	22.0 ± 1.0	18.0 ± 3.6	5.90 ± 1.21	4.97 ± 0.67 ^b^	15.1 ± 0.5
2018	23.6 ± 1.1	15.0 ± 0.7	6.93 ± 0.46	8.50 ± 1.15 ^a^	22.2 ± 0.6
Weiki					
2015	41.8 ± 4.6	20.6 ± 1.1 ^a^	13.27 ± 2.50	6.84 ± 0.71 ^b^	17.4 ± 0.7
2016	35.1 ± 12.3	13.6 ± 2.4 ^b^	7.17 ± 1.18	6.03 ± 1.15 ^b^	13.8 ± 0.7
2018	28.7 ± 2.2	10.3 ± 0.1 ^b^	8.22 ± 0.42	9.27 ± 0.98 ^a^	21.6 ± 0.4
Bingo					
2015	23.0 ± 0.6	9.2 ± 0.4	9.12 ± 0.56	4.35 ± 0.53	16.5 ± 0.6
2016	23.1 ± 4.3	9.1 ± 5.3	7.87 ± 4.56	4.47 ± 1.51	14.1 ± 0.7
2018	18.6 ± 0.9	8.3 ± 2.7	6.38 ± 0.65	4.35 ± 0.88	21.1 ± 3.0
Anna					
2015	32.1 ± 2.4	22.1 ± 2.0 ^a^	12.30 ± 0.10	5.80 ± 0.45 ^b^	17.0 ± 0.5
2016	22.9 ± 3.8	9.6 ± 5.0 ^b^	5.33 ± 2.68	5.87 ± 1.67 ^b^	14.0 ± 0.8
2018	25.6 ± 1.7	14.2 ± 3.8 ^b^	8.11 ± 0.96	9.97 ± 1.30 ^a^	22.5 ± 0.6
Global cultivar effect (2015, 2016, and 2018)					
Geneva	23.5 ± 1.5 ^B^	15.4 ± 2.9 ^A^	7.60 ± 2.12	6.30 ± 1.81 ^A^	17.6 ± 3.5
Weiki	35.2 ± 8.7 ^A^	14.8 ± 4.8 ^A^	9.55 ± 3.15	7.38 ± 1.68 ^A^	17.6 ± 3.4
Bingo	21.6 ± 3.1 ^B^	8.8 ± 3.0 ^B^	7.79 ± 2.61	4.39 ± 0.92 ^B^	17.3 ± 3.5
Anna	26.9 ± 4.8 ^B^	15.3 ± 6.4 ^A^	8.58 ± 3.35	7.21 ± 2.33 ^A^	17.8 ± 3.8
Year effect					
2015	30.4 ± 8.0 ^A^	16.3 ± 5.7 ^A^	11.16 ± 2.19 ^A^	5.60 ± 1.04 ^B^	16.6 ± 0.9 ^B^
2016	25.8 ± 8.1 ^AB^	12.6 ± 5.2 ^B^	6.57 ± 2.59 ^B^	5.33 ± 1.30 ^B^	14.3 ± 0.8 ^C^
2018	24.1 ± 4.1 ^B^	11.9 ± 3.5 ^B^	7.41 ± 0.99 ^B^	8.02 ± 2.46 ^A^	21.9 ± 1.5 ^A^
Interaction	Ns	<0.01	Ns	<0.05	Ns

In each subcolumn, data marked with different lowercase or uppercase superscript letters differ significantly at *p* ≤ 0.05 according to the post hoc pairwise comparisons with Tukey adjustment for the familywise error.

## Data Availability

Not applicable.

## Supplementary Materials

The following supporting information can be downloaded at: https://www.mdpi.com/article/10.3390/molecules28010455/s1, Figure S1: A graph of the Pearson correlation matrix (correlogram) of the morphological, chemical, and antioxidant properties of the fruits. The significance level is equal to 0.1.

## Sample Availability

Samples of the compounds are not available from the authors.

## References

[1] Oz A.T., Kafkas E., Waisundara V., Shiomi N. (2017). Phytochemicals in Fruits and Vegetables. Superfood and Functional Food—An Overview of Their Processing and Utilization.

[2] Yeshi K., Crayn D., Ritmejerytė E., Wangchuk P. (2022). Plant Secondary Metabolites Produced in Response to Abiotic Stresses Has Potential Application in Pharmaceutical Product Development. Molecules.

[3] Zhbanova Е.V., Lukyanchuk I.V. (2021). Variability of the chemical composition of fruits of strawberry selected hybrid forms. Pomic. Small Fruits Cult. Russ..

[4] Cossio F., Debersaques F., Latocha P. (2015). Kiwiberry (*Actinidia arguta*): New perspectives for a great future. Acta Hortic..

[5] Latocha P., Vereecke D., Debersaques F. (2018). Kiwiberry Commercial Production—What Stage Are We at?. Acta Hortic..

[6] Bieniek A., Dragańska E., Pranckietis V. (2016). Assessment of climatic conditions for *Actinidia arguta* cultivation in north-eastern Poland. Zemdirbyste.

[7] Latocha P. (2017). The Nutritional and Health Benefits of Kiwiberry (*Actinidia arguta*)—A Review. Plant Foods Hum. Nutr..

[8] Pinto D., Delerue-Matos C., Rodrigues F. (2020). Bioactivity, phytochemical profile and pro-healthy properties of Actinidia arguta: A review. Food Res. Int..

[9] Leontowicz M., Leontowicz H., Jesion I., Bielecki W., Najman K., Latocha P., Park Y.-S., Gorinstein S. (2016). *Actinidia arguta* supplementation protects aorta and liver in rats with induced hypercholesterolemia. Nutr. Res..

[10] Błaszczak W., Latocha P., Jeż M., Wiczkowski W. (2021). The impact of high-pressure processing on the polyphenol profile and anti-glycaemic, anti-hypertensive and anti-cholinergic activities of extracts obtained from kiwiberry (*Actinidia arguta*) fruits. Food Chem..

[11] Fisk C.L., Mc Daniel M.R., Strik B.C., Zhao Y. (2006). Physicochemical, sensory, and nutritive qualities of hardy kiwifruit (*Actinidia arguta* ‘Ananasnaya’) as affected by harvest maturity and storage. J. Food Sci..

[12] Marangi F., Pinto D., de Francisco L., Alves R.C., Puga H., Sut S., Dall’Acqua S., Rodrigues F., Oliveira M.B.P.P. (2018). Hardy kiwi leaves extracted by multi-frequency multimode modulated technology: A sustainable and promising by-product for industry. Food Res. Int..

[13] Silva A.M., Costa P., Delerue-Matos C., Latocha P., Rodrigues F. (2021). Extraordinary composition of *Actinidia arguta* by-products as promising skin ingredient: A new challenge for cosmetic industry. Trends Food Sci. Technol..

[14] Park Y.-S., Im M.H., Ham K.-S., Kang S.-G., Park Y.-K., Namiestnik J., Leontowicz H., Leontowicz M., Katrich E., Gorinstein S. (2011). Nutritional and Pharmaceutical Properties of Bioactive Compounds in Organic and Conventional Growing Kiwifruit. Plants Foods Hum. Nutr..

[15] Wojdyło A., Nowicka P., Oszmiański J., Golis T. (2017). Phytochemical compounds and biological effects of Actinidia fruits. J. Funct. Foods.

[16] Nishiyama I., Yamashita Y., Yamanaka M., Shimohashi A., Fukuda T., Oota T. (2004). Varietal difference in vitamin C content in the fruit of kiwifruit and other *Actinidia* species. J. Agric. Food Chem..

[17] Leontowicz H., Leontowicz M., Latocha P., Jesion J., Park Y.-S., Katrich E., Barasch D., Nemirovski N., Gorinstein S. (2016). Bioactivity and nutritional properties of hardy kiwi fruit *Actinidia arguta* in comparison with *Actinidia deliciosa* ‘Hayward’ and *Actinidia eriantha* ‘Bidan’. Food Chem..

[18] Bieniek A. (2012). Mineral composition of fruits of *Actinidia arguta* and *Actinidia purpurea* and some of their hybrid cultivars grown in north-eastern Poland. Pol. J. Environ. Stud..

[19] Drzewiecki J., Latocha P., Leontowicz H., Leontowicz M., Park Y.S., Najman K., Weisz M., Ezra A., Gorinstein S. (2016). Analytical methods applied to characterization of *Actinidia arguta*, *Actinidia deliciosa* and *Actinidia eriantha* kiwi fruit cultivars. Food Anal. Methods.

[20] Stefaniak J., Przybył J., Latocha P., Łata B. (2020). Bioactive compounds, total antioxidant activity and yield of kiwiberry fruit under different nitrogen regimes in field conditions. J. Sci. Food Agric..

[21] Kim J.G., Beppu K., Kataoka I. (2009). Varietal differences in phenolic content and astringency in skin and flesh of hardy kiwifruit resources in Japan. Sci. Hortic..

[22] Latocha P., Łata B., Stasiak A. (2015). Phenolics, ascorbate and the antioxidant potential of kiwiberry vs. common kiwifruit: The effect of cultivar and tissue type. J. Funct. Foods.

[23] Richardson A.C., Marsh K.B., Boldingh H.L., Pickering A.H., Bulley S.M., Frearson N.J., Ferguson A.R., Thornber S.E., Bolitho K.M., Macrae E.A. (2004). High growing temperatures reduce fruit carbohydrate and vitamin C in kiwifruit. Plant Cell Environ..

[24] Nishiyama I., Fukuda T., Oota T. (2005). Genotype differences in chlorophyll, lutein, and b-carotene contents in the fruits of Actinidia species. J. Agric. Food Chem..

[25] Latocha P., Wołosiak R., Worobiej E., Krupa T. (2013). Clonal differences in antioxidant activity and bioactive constituents of hardy kiwifruit (*Actinidia arguta*) and its year-to-year variability. J. Sci. Food Agric..

[26] Łata B., Przeradzka M., Bińkowska M. (2005). Great differences in antioxidant properties exist between 56 apple cultivars and vegetation seasons. J. Agric. Food Chem..

[27] Latocha P., Debersaques F., Decorte J. (2015). Varietal Differences in the Mineral Composition of Kiwiberry—*Actinidia arguta* (Siebold et Zucc.) Planch. ex. Miq. Acta Hortic..

[28] Gralak M.A., Lasocka I., Leontowicz M., Leontowicz H., Latocha P., Gorinstein S. (2022). Bioavailability of Macro- and Microelements in Rats Fed Hypercholesterolemic Diets Containing *Actinidia arguta* Fruits. Foods.

[29] Bieniek A., Dragańska E. (2013). Content of macroelements in fruits of Ukrainian cultivars of Hardy kiwifruit and Actinidia charta depending on the weather conditions during the phonological phases. J. Elem..

[30] Gündüz K., Serҫe S., Hancock J.F. (2015). Variation among highbush and rabbiteye cultivars of blueberry for fruit quality and phytochemical characteristics. J. Food Compos. Anal..

[31] Swoczyna T., Łata B., Stasiak A., Stefaniak J., Latocha P. (2019). JIP-test in assessing sensitivity to nitrogen deficiency in two cultivars of *Actinidia arguta* (Siebold et Zucc.) Planch. ex Miq. Photosynthetica.

[32] Stefanelli D., Goodwin I., Jones R. (2010). Minimal nitrogen and water use in horticulture: Effects on quality and content of selected nutrients. Food Res. Int..

[33] Stefaniak J., Stasiak A., Latocha P., Łata B. (2019). Seasonal changes in macronutrients in the leaves and fruit of kiwiberry: Nitrogen level and cultivar effects. Commun. Soil Sci. Plant Anal..

[34] Osborne B.A., Raven J.A. (1986). Light absorption by plants and its implications for photosynthesis. Biol. Rev. Camb. Philos. Soc..

[35] Sid’ko A.F., Botvich I.Y., Pis’man T.I., Shevyrnogov A.P. (2017). Estimation of the chlorophyll content and yield of grain crops via their chlorophyll potential. Biophysics.

[36] Gholamin R., Khayatnezhad M. (2011). The effect of end season drought stress on the chlorophyll content, chlorophyll fluorescence parameters and yield in maize cultivars. Sci. Res. Essays.

[37] Ghosh M., Pal A.K., Pal S.K., De D.K. (2003). Relationship of leaf area and chlorophyll content with yield of aromatic rice. Indian J. Plant Physiol..

[38] Smith G.S., Buwalda J.G., Clark C.J. (1988). Nutrient dynamics of a kiwifruit ecosystem. Sci. Hortic..

[39] Ludemann C.I., Gruere A., Heffer P., Dobermann A. (2022). Global data on fertilizer use by crop and by country. Sci. Data.

[40] Zhao Z., Tong Y., Wang J. (2013). Nutrient Uptake and Distribution in Field-Grown Kiwifruit Vines. Acta Hortic..

[41] Tränknera M., Ershad Tavakol E., Jákli B. (2018). Functioning of potassium and magnesium in photosynthesis, photosynthate translocation and photoprotection. Physiol. Plant..

[42] Yruela I. (2005). Copper in plants. Braz. J. Plant Physiol..

[43] Brown P.H., Bellaloui N., Wimmer M.A., Bassil E.S., Ruiz J., Hu H., Pfeffer H., Dannel F., Römheld V. (2002). Boron in Plant Biology. Plant Biol..

[44] Palmer J.W., Giuliani R., Adams H.M. (1997). Effect of crop load on fruiting and leaf photosynthesis of ‘Braeburn’/M.26 apple trees. Tree Physiol..

[45] Sidhu R.S., Bound A.A., Hunt I. (2022). Crop Load and Thinning Methods Impact Yield, Nutrient Content, Fruit Quality, and Physiological Disorders in ‘Scilate’ Apples. Agronomy.

[46] Testolin R. (1990). Kiwifruit yield efficiency, plant density, and bud number per surface unit. J. Am. Soc. Hortic. Sci..

[47] Kopsell D.A., Kopsell D.E., Lefsrud M.G. (2004). Variation in lutein, β-carotene, and chlorophyll concentrations among Brassica oleraceae cultings and seasons. HortScience.

[48] Ihl M., Shene C., Scheuermann E., Bifani V. (2006). Correlation of pigment content through colour determination using tristimulus values in a green leafy vegetable, Swiss chard. J. Sci. Food Agric..

[49] Mou B. (2005). Genetic Variation of Beta-carotene and Lutein contents in Lettuce. J. Am. Soc. Hortic. Sci..

[50] Holasová M., Dostálová R., Fiedlerová V., Horáček J. (2009). Variability of Lutein Content in Peas (*Pisum sativum* L.) in Relation to the Variety, Season and Chlorophyll Content. Czech J. Food Sci..

[51] Delgado-Pelayo R., Gallardo-Guerrero L., Hornero-Méndez D. (2014). Chlorophyll and carotenoid pigments in the peel and flesh of commercial apple fruit varieties. Food Res. Int..

[52] Strik B.C., Davis A.J. (2021). Growing Kiwifruit. A Guide to Kiwiberries and Fuzzy Kiwifruit for Pacific Northwest Producers, PNW 507.

[53] Komosa A., Roszyk J., Mieloch M. (2017). Content of nutrients in soils of highbush blueberry (*Vaccinium corymbosum* L.) plantations in Poland in a long-term study. J. Elem..

[54] Escarpa A., Gonzalez M.C. (1998). High-performance liquid chromatography with diode-array detection for the determination of phenolic compounds in peel and pulp from different apple varieties. J. Chromatogr. A.

[55] Medina M.B. (2011). Determination of the total phenolics in juices and superfruits by novel chemical method. J. Funct. Foods.

[56] Łata B. (2014). Variability in enzymatic and non-enzymatic antioxidants in red and green-leafy kale in relation to soil type and N-level. Sci. Hortic..

[57] Re R., Pellegrini N., Proteggente A., Pannala A., Yang M., Rice-Evans C. (1999). Antioxidant activity applying an improved ABTS radical cation decolorization assay. Free. Radic. Biol. Med..

[58] Brand-Williams W., Cuvelier M.E., Berset C. (1995). Use of a free radical method to evaluate antioxidant activity. LWT Food Sci. Technol..

[59] R Core Team R (2022). A Language and Environment for Statistical Computing.

