# Supplementary material for: Variation of Chemical Composition and Antioxidant Properties of Kiwiberry (Actinidia arguta) in a Three-Year Study
Source: Molecules. 2023 Jan 3;28(1):455. doi: 10.3390/molecules28010455 (PMC9823446; doi:10.3390/molecules28010455)
Supplement: Supplementary file 1 [file molecules-28-00455-s001.zip › molecules-2113432-supplementary.pdf]

Supplementary Material

Variation of Chemical Composition and Antioxidant Properties of Kiwiberry (*Actinidia arguta*) in a Three-Year Study

Piotr Latocha [1,*], Barbara Łata [2] and Paweł Jankowski [3]

[1] Department of Environmental Protection and Dendrology, Institute of Horticultural Sciences Warsaw University of Life Sciences—SGGW, Nowoursynowska 166, 02-787 Warsaw, Poland
[2] Section of Basic Research in Horticulture, Institute of Horticultural Sciences Warsaw University of Life Sciences—SGGW, Nowoursynowska 166, 02-787 Warsaw, Poland
[3] Department of Computer Information Systems, Institute of Information Technology, Warsaw University of Life Sciences—SGGW, Nowoursynowska 166, 02-787 Warsaw, Poland

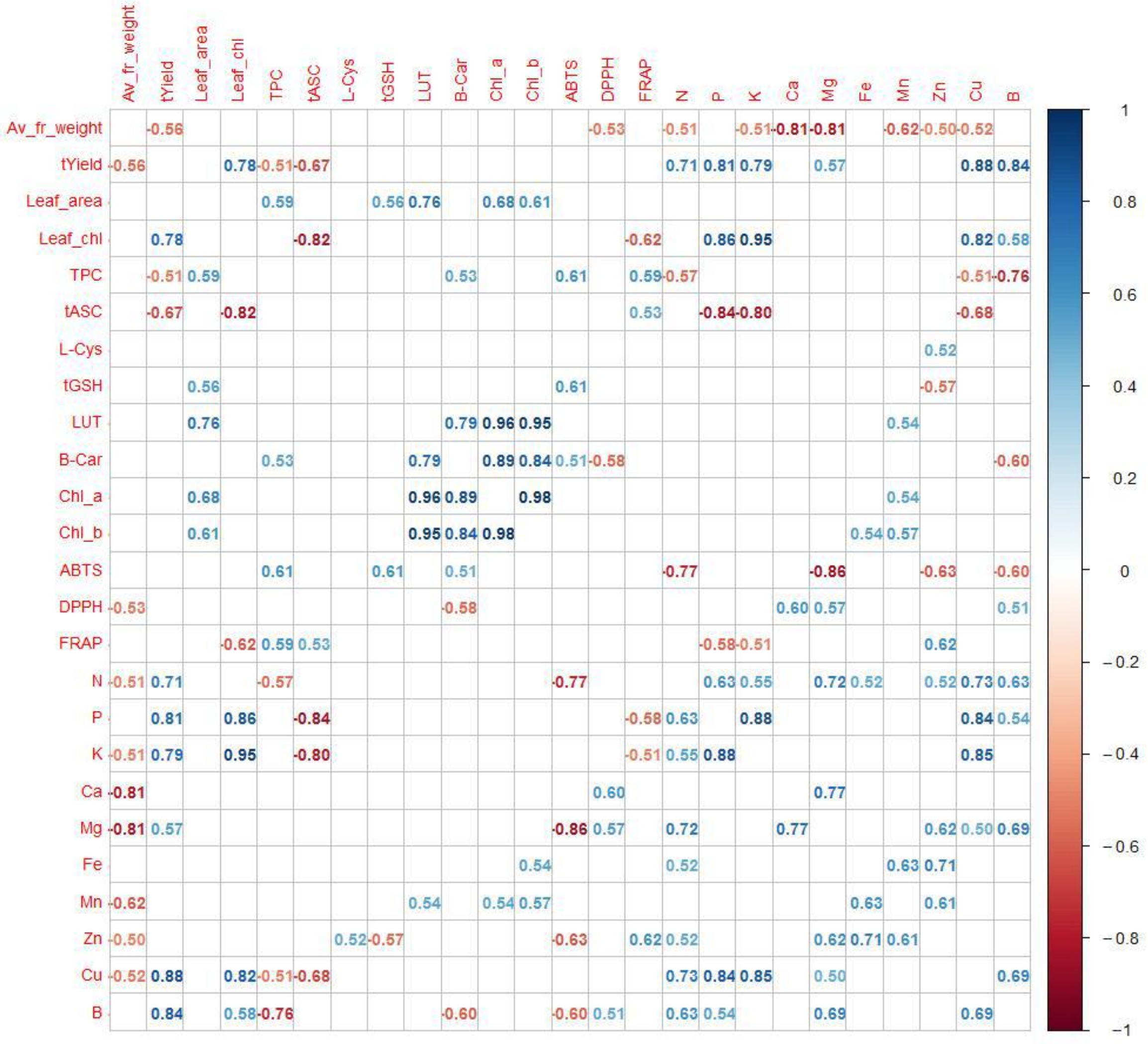

Figure S1. A graph of the Pearson correlation matrix (correlogram) of the morphological, chemical, and antioxidant properties of the fruits. The significance level equal to 0.1. The blue colour represent the positive correlations and the red colour the negative ones. Each number shows a value of the significant correlation. The colour intensity represents the correlation strength, according to the legend.
